# Depression and Temporal Lobe Epilepsy: Expression Pattern of Calbindin Immunoreactivity in Hippocampal Dentate Gyrus of Patients Who Underwent Epilepsy Surgery with and without Comorbid Depression

**DOI:** 10.1155/2019/7396793

**Published:** 2019-05-02

**Authors:** Luciana D'Alessio, Hector Konopka, Patricia Solís, Laura Scévola, Mónica Fernandez Lima, Claudio Nuñez, Eduardo Seoane, Silvia Oddo, Silvia Kochen

**Affiliations:** ^1^ENyS-IBCN, Neuroscience and Systems Institute and Cell Biology and Neuroscience Institute, CONICET, National Council for Scientific and Technical Research of Argentina, Universidad de Buenos Aires, Buenos Aires, Argentina; ^2^Epilepsy Center of Ramos Mejía and El Cruce Hospitals, Buenos Aires, Argentina

## Abstract

**Purpose:**

Changes in calbindin (CB) expression have been reported in patients with temporal lobe epilepsy (TLE) with controversial implications on hippocampal functions. The aim of this study was to determine the CB immunoreactivity in hippocampal dentate gyrus of patients who underwent epilepsy surgery for drug-resistant TLE with and without comorbid depression and/or memory deficits.

**Methods:**

Selected hippocampal samples from patients with TLE who underwent epilepsy surgery were included. Clinical and complementary assessment: EEG, video-EEG, MRI, psychiatric assessment (structured clinical interview, DSM-IV), and memory assessment (Rey auditory verbal learning test, RAVLT; Rey-Osterrieth complex figure test, RCFT), were determined before surgery. Hippocampal sections were processed using immunoperoxidase with the anti-calbindin antibody. The semiquantitative analysis of CB immunoreactivity was determined in dentate gyrus by computerized image analysis (ImageJ).

**Results:**

Hippocampal sections of patients with TLE and HS (*n* = 24) and postmortem controls (*n* = 5) were included. A significant reduction of CB+ cells was found in patients with TLE (*p* < 0.05, Student's *t*-test). Among TLE cases (*n* = 24), depression (*n* = 12) and memory deficit (*n* = 17) were determined. Depression was associated with a higher % of cells with the CB dendritic expression (CB-sprouted cells) (*F*(1, 20) = 11.81, *p* = 0.003, hp^2^ = 0.37), a higher CB+ area (*μ*m^2^) (*F*(1, 20) = 5.33, *p* = 0.032, hp^2^ = 0.21), and a higher optical density (*F*(1, 20) = 15.09, *p* = 0.001, hp^2^ = 0.43) (two-way ANOVA). The GAF scale (general assessment of functioning) of DSM-IV inversely correlated with the % of CB-sprouted cells (*r* = −0.52, *p* = 0.008) and with the CB+ area (*r* = −0.46, *p* = 0.022).

**Conclusions:**

In this exploratory study, comorbid depression was associated with a differential pattern of CB cell loss in dentate gyrus combined with a higher CB sprouting. These changes may indicate granular cell dysmaturation associated to the epileptic hyperexcitability phenomena. Further investigations should be carried out to confirm these preliminary findings.

## 1. Introduction

Depression disorders are the most common psychiatric diagnosis in persons with drug-resistant temporal lobe epilepsy (TLE). The prevalence rate oscillates between 30 and 35% in population-based studies, reaching the highest prevalence (50%) at specialized epilepsy centers [1]. Depression disorders have been associated with a worse quality of life and a poorer treatment response to both pharmacological and surgical treatments [2–6]. Additionally, population-based studies described that a positive history of depression was more frequently found among patients with epilepsy onset indicating a bidirectional relationship [7].

The complex underlying pathogenic mechanisms of this association are still unknown; however, it has been proposed that epileptic phenomena may negatively affect dentate gyrus granular cell neuroplasticity, with implications on hippocampal-dependent functions, facilitating cognitive impairments (episodic memory) and emotional alterations (depression and stress vulnerability) [8–12]. Hippocampal sclerosis (HS) is the most frequent (60-70%) neuropathological finding in patients with drug-resistant TLE and is characterized by a loss of pyramidal neurons in CA1-4 of the hippocampus. Dentate gyrus layers are also affected in a variable proportion of patients ranging from 50% to 80% in patients with chronic epilepsy, with even higher rates reaching 100% in reports from TLE patients with hippocampal sclerosis [13, 14]. Dentate gyrus pathology is characterized by neuronal loss, granular cell dispersion, ectopic localization, aberrant synapsis, and abnormal sprouting of mossy fibers [13–17]. Furthermore, a reduction of dentate gyrus neuroplasticity with lower rates of newly formed cells has been found in experimental models of chronic epilepsy [18–20] and in human patients with drug-resistant epilepsy [21–24]. In this line, in the previous studies performed by our group, we found a reduction of the newly formed cell neuronal markers, doublecortin and nestin, in dentate gyrus from adult patients with chronic TLE with comorbid psychiatric and cognitive disorders [25, 26].

Calbindin is a calcium-binding protein preferentially expressed in excitatory granular cells of dentate gyrus layers and constitutes a marker of granule cell maturity [27–29]. The reduction of the calbindin (CB) (28 kD) expression in dentate gyrus constitutes an early and common morphological sign observed in animal models of epilepsy [30, 31] and in human TLE [27, 28, 32, 33] with and without hippocampal sclerosis [34]. It has been observed in the animal models that both situations, CB deficiency and CB overexpression in dentate gyrus, may reduce LTP (long-term potentiation) and memory [35, 36]. Studies of the calbindin expression in the dentate gyrus from patients with TLE and hippocampal sclerosis qualitatively described different patterns [17, 33]: the pattern 1, characterized by a reduced number of CB+ granular cells predominantly among basal layers with a higher proportion of strong CB+ cells located in outer layers, with CB-positive sprouted fibers and ectopic localization (inner molecular layers); the pattern 2 (normal) observed in normal postmortem samples and in some TLE patients, characterized by a homogeneous pattern; and the pattern 3 with a homogeneous reduction of CB+ granular cells.

Only a few studies determined the clinical and pathological correlations between CB alterations and memory in patients with epilepsy with controversial results [32, 33]; however, how these alterations are related with emotional disturbances and depression has not been described. The aim of this study was to analyze the expression of CB immunoreactivity, a marker of mature granular cells. We studied the hippocampal dentate gyrus layers of patients with drug-resistant TLE who underwent epilepsy surgery, with and without comorbid psychiatric depression and/or memory deficits before epilepsy surgery.

## 2. Methods

### 2.1. Study Design and Patient Selection

This study was performed at the Epilepsy Center of the Ramos Mejía Hospital and El Cruce Hospitals and at the Institutes ENyS-IBCN-CONICET, Buenos Aires, Argentina. The Epilepsy Center is the main reference public center in the country for epilepsy surgery and assists a population with the high rates of drug-resistant epilepsy (70%-80%) [37, 38].

This study was conducted with the approval of the Ethics Committee of Ramos Mejía and EL Cruce Hospitals, in accordance with the ethical standards laid down in the 1964 Declaration of Helsinki, and all the subjects' submitted the informed consent for participating in the study.

#### 2.1.1. Inclusion Criteria

We selected hippocampal samples obtained from patients who underwent epilepsy surgery for temporal lobe drug-resistant epilepsy (TLE) [14]. Only patients who had completed the psychiatric assessment protocol and neuropsychological assessment before surgery and who signed the approved informed consent for participation were included [39, 40].

Selected samples from patients with TLE were grouped according to categorical variables: (1) depression factor: it was considered positive when patient met the criteria for at least one current or past interictal episode of major depression and/or other depression disorders, codified in axis I of DSM-IV using SICD I (dysthymia, major depression with or without psychotic symptoms, and/or recurrent depression). (2) Memory factor: memory was pondered impaired when Rey auditory verbal learning test (RAVLT) Spanish version and/or Rey-Osterrieth complex figure test (RCFT) scores were >2 of standard deviation from normal data adjusted by age and gender according to Lezak [41].

#### 2.1.2. Exclusion Criteria

The exclusion criteria were the present or past history of other chronic interictal psychiatric disorders (nonaffective disorders) codified in other sections in axis I of DSM-IV (i.e., chronic psychosis) and patients with mental retardation (IQ < 70 and/or attendance to a special school).

### 2.2. Diagnosis of Drug-Resistant TLE

Prior to surgery, all patients were evaluated according to the Epilepsy Center diagnosis protocol, specially designed for patients with resistant epilepsy and surgical candidates. This protocol includes the clinical examination by trained neurologists and complementary studies to confirm the temporal lobe origin of the epileptogenic zone by interictal EEG, video-EEG monitoring, magnetic resonance imaging (MRI) with a temporal lobe epilepsy protocol, and neuropsychological assessment [37, 38].

### 2.3. Video-EEG Evaluation

All patients included in this study underwent video-EEG evaluation in order to confirm the epileptogenic zone and to determine the possibility of epilepsy surgery. For long-term EEG monitoring, a Stellate-Bioscience EEG machine at a 200 Hz sample rate was used. All ictal recordings were obtained using the international 10–20 system with the addition of temporal electrodes of the 10–10 system. Referential montages as well as longitudinal bipolar and transverse bipolar montages were used for the analysis.

### 2.4. Magnetic Resonance Image (MRI)

Magnetic resonance image protocol used was sagittal T1-weighted fluid-attenuated inversion recovery (FLAIR) T1 FFE 3D acquisition, perpendicular to the long axis of the hippocampus, and T2-weighted axial, parallel to the long axis of the hippocampus. All patients included in this study met the image criteria for HS diagnosis: atrophy, hypointense in T1W and IR, hyperintense in T2W and FLAIR, and alteration of the internal structure of the hippocampus [42].

### 2.5. Neuropsychological Assessment

All patients underwent a neuropsychological assessment protocol before surgery (usually 6 months to one year before surgery), provided by trained specialists. In this study, verbal memory was determined using the Rey auditory verbal learning test (RAVLT), Spanish version [43]. It consists of reading a list of words in five different trials, recovering the immediate memory and differed memory, and recognition in each trial. For visual memory, the Rey-Osterrieth complex figure test (RCFT) was determined. This nonverbal test consists of presenting a visual design that patients had to copy first and reproduce immediately after the visual presentation (immediate recall) and after 30 minutes (differed recall). Considering there is no regional normative data for Argentinian population, international data was used to compare our results [41].

### 2.6. Psychiatric Assessment

All patients included in this study underwent a complete psychiatric assessment prior to surgery (usually 6 months to one year before surgery). Psychiatric assessment was performed routinely by trained psychiatrists according to a standardized protocol specially designed for patients with epilepsy [40, 44]. Psychiatric history was obtained from each patient and relatives, complemented by information from relatives. The psychiatric semiology of the witnessed examination was supplemented with the structured clinical interview (SCID) Spanish version for DSM-IV axis I diagnoses SCID-I and SCID II for personality disorders [45, 46]. Additionally, all patients were assessed according the global assessment of functioning (GAF) of DSM-IV and to the Beck depression scale. The GAF is a 100-point tool rating overall psychological, social, and occupational functioning in relation to psychiatric assessment, included in the DSM-IV in the section on multiaxial assessments (axis V of DSM-IV) [47]. The interviews were carried out in approximately 2 to 3 hours. Beck depression inventory II, Spanish version, was also administered to quantify depression symptoms (33). Beck inventory was added to the protocol at the Epilepsy Center after 2010, and only the patients included in the last period had completed this item prospectively.

### 2.7. Tissue Processing and Pathological Diagnoses

The surgical piece was fixed in formalin for 7 days. Tissue blocks (thickness: 5 mm) were made following coronal planes and were embedded in paraffin for storage. For this study, completed coronal hippocampal sections localized at the anterior medial region of the hippocampus body were cut at 7 *μ*m with a microtome, stretched in water, mounted on slides, deparaffined in xylene, and hydrated and stained with haematoxylin-eosin and thionin stain. Hippocampal samples were studied by a trained neuropathologist with routine techniques including haematoxylin-eosin and thionin stain, and additionally, some samples were stained with immunohistochemistry with anti-NeuN and anti-GFAP to determine the pathological diagnosis [14]. Archival material of the normal postmortem hippocampus donated from autopsies to the pathology department for research was included. Samples were selected and matched by gender and age, and the sample free from neurological injury, drug and/or alcohol dependency, and suicidal evidences was simultaneously processed as controls.

#### 2.7.1. Immunohistochemistry

After deparaffinization, sections were treated according to the following procedure: a 15 min wash in distilled water, then an incubation in a microwave at 100°C twice for 5 min in a citric acid solution (0.1 mol/L citric acid monohydrate and 0.1 mol/L trisodium citrate dihydrate), pH 6.0, and after that a 2-fold 5 min wash in phosphate-buffered saline (PBS); the sections were incubated for 30 min in 0.5% (*v*/*v*) hydrogen peroxide in ethanol to quench endogenous peroxidases. Afterwards, they were incubated overnight in a humid chamber at 4°C with rabbit polyclonal anti-calbindin (28 kD) Millipore cat # AB1778, 1/500 in PBS Triton X-100, and 0.1% (*w*/*v*) sodium azide. Additionally, other sections were incubated with the mouse monoclonal antibody, anti-NeuN Millipore cat # MAB377 1/200, and others with rabbit polyclonal anti-GFAP Dako (1/200) cat # 300742EFG. After that, a biotinylated secondary anti-mouse/rabbit antibody (1 : 1000, Vector Laboratories) followed by 30 min in Vectastain Elite ABC solution (Vector Laboratories) was incubated at room temperature. The complex was detected using the supersensitive multilink-HRP/DAB kit from Bio-Genex cat # QD000-5L following the vendor's procedure, and haematoxylin staining was added at the end of the procedure. Controls omitting the primary and the secondary antibody were determined [26].

#### 2.7.2. Microscope Image Analysis

Sections immunostained with CB were examined in detail for each case. The images were acquired by a SONY Power HAD 3CCD colour video camera system from a Zeiss Axiophot microscope. Microphotograph images (20x) were captured from different areas along the transversal axis of hippocampal dentate gyrus and were digitalized with a resolution of 1280-960 pixels. All images (five images per case) were captured under identical lighting and magnification conditions. The semiquantitative measurements of the microphotographs including all dentate gyrus layers by field (molecular layer, granular cell layer, and polymorphic layer including those granular cells dispersed in the molecular layer) were determined. The semiquantitative examination of the CB staining in dentate gyrus was determined by single-cell analysis using the computerized image analysis program, ImageJ. Two independent and blinded observers made the measures. The parameters analyzed were the following: (a) calbindin-positive cells (CB+) and CB-negative cells (CB-), (CB- was considered for haematoxylin-positive nucleus) by field were counted. The proportion of CB+ cells was expressed as the percentage of the entire granule cell population (CB+ and CB-) counted by field. (b) The mean of CB+ reactive surface (reactive area of soma) was determined for CB+ cells (*μ*m^2^). (c) The staining intensity (optical density) was calculated using the mean gray value (MGV) function of the ImageJ. It was calculated for each CB+ cell (soma), and the background was subtracted (inverted measures). The MGV of the background was calculated using 3 measures by photo. The final result indicates a direct measure of the intensity of the immunoreaction. (d) The proportion of CB+ cells with soma and dendritic CB expression (sprouted cells) was expressed as a percentage of the entire CB+ cells counted. (e) The mean length of the CB+ dendritic processes was measured (CB+ processes emerging from the soma > 2 *μ*m of length were considered). The parameters described in the point (b) and point (c) were only analyzed in TLE samples, since the postmortem period with different times of fixation may modify the results.

#### 2.7.3. Statistical Analysis

Normality distribution was determined using the Shapiro-Wilk test. Two-tailed significant levels were fixed at *p* < 0.05, (1-*β*power ≥ 0.80). Descriptive statistics were performed, and the chi-square test was used to analyze qualitative variables. The Student *t*-test and the two-way ANOVA were applied when normal distribution was found and nonparametric tests (Mann-Whitney) were applied when distribution was not normal (Shapiro-Wilk test < 0.05). The effect size was calculated using partial eta squared (hp^2^), and the presence of significative interactions was determined. Pearson correlation coefficients were analyzed among quantitative variables. A computational program for Windows (SPSS 22 version) was used to perform the statistical analysis.

## 3. Results

Hippocampal sections of the selected patients with TLE and HS consecutively operated between 2006 and 2014 (*n* = 24) and postmortem controls (*n* = 5) were included. Among TLE cases (*n* = 24), depression (*n* = 12) and memory deficit (*n* = 17) were determined during the presurgical assessment protocol. Memory deficits were presented in 66% (*n* = 8) of patients with TLE and depression and in 75% (*n* = 9) among patients with TLE without depression (*p* = 0.65, chi-square test). Demographic, clinical, psychiatric, neuropsychological, and neuropathological data of patients are shown in Table 1.

Archival material obtained from the normal postmortem hippocampus matched by gender and age, free from neurological injury, drug, and/or alcohol dependency, was simultaneously processed as controls. The time of autopsy never exceeded 6 hours. Five postmortem controls (2 male, 3 female, mean age 41 ± 18) (*p* > 0.05, chi-square test comparing age and sex with the TLE group) were included. The causes of death were cardiopathy (*n* = 2) and nonencephalic trauma (*n* = 3).

**Figure 1:**
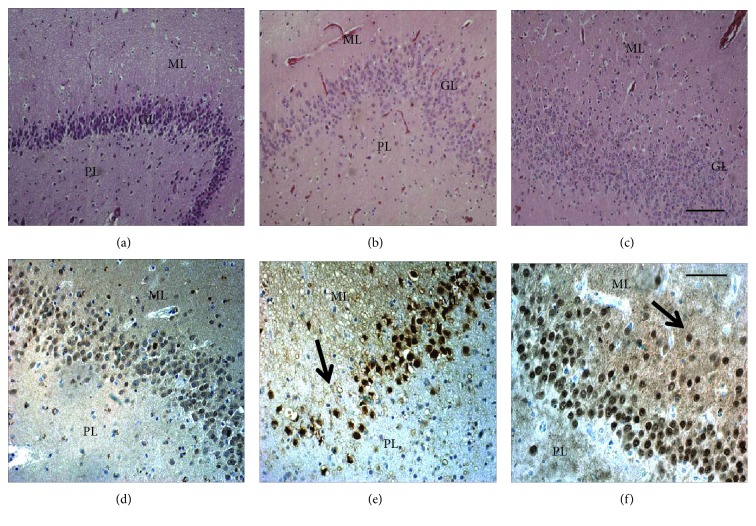
Hippocampal dentate gyrus from controls and patients with TLE and hippocampal sclerosis.

**Figure 2:**
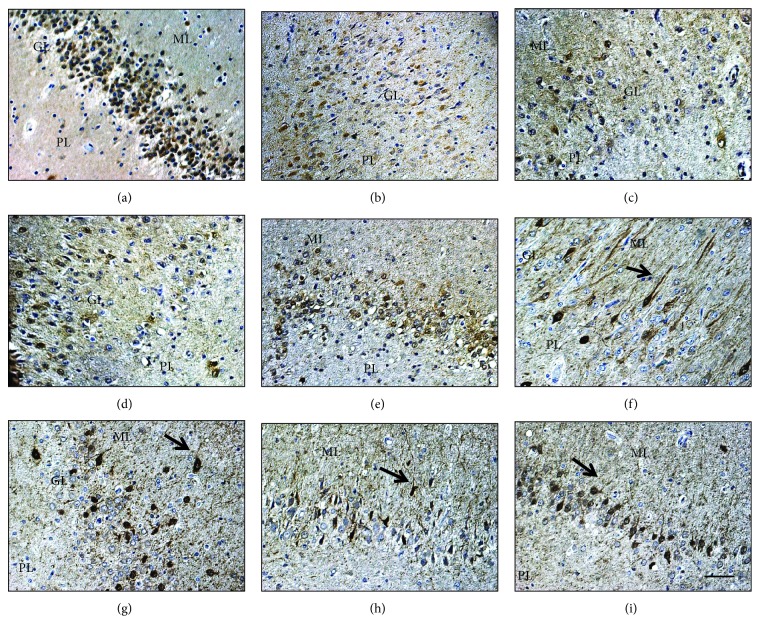
Hippocampal sections from postmortem control (a) and TLE with HS (b–i). PL: polymorphic layer; ML: molecular layer; GL: granular cell layer. (a) Qualitative analysis of postmortem control shows homogeneous CB+ granular cell morphology and staining and the absence of GL dispersion and/or ectopic localization. (b–e) Qualitative examination of dentate gyrus sections from TLE patients showed lower CB+ cell density among all dentate gyri (cases 5, 6, 14, and 15 of Table 1). (f–i) Qualitative examination of dentate gyrus sections from TLE+D patients showed a pattern with a reduced number of CB+ granular cells predominantly among basal layers (basal granule cells are predominantly CB negative), with a great proportion of strong CB+ cells located in outer layers, with CB-positive arborization (CB+ sprouted cells), ectopic localization (inner molecular layers), higher CB+ content (higher staining intensity and CB+ area), and longer apical CB+ sprouted fibers projecting into ML (arrows). Basal CB+ sprouted processes are also observed (cases 20, 21, 23, and 24 of Table 1). Magnification (20x), scale bar 30 *μ*m.

Figure 1 shows dentate gyrus from postmortem controls and TLE patients, viewing dentate gyrus dispersion and cell loss in TLE patients.  Figure 2 shows dentate gyrus CB immunoreactivity and the different patterns described by Martinian et al. [33]. Qualitative examination of dentate gyrus patterns showed a normal pattern (pattern 2) in postmortem controls (Figure 2(a)), a lower CB+ cell density among dentate gyrus layers (pattern 3) in TLE patients (Figures 2(b)–2(e)), and a reduced number of CB+ granular cells predominantly among basal layers with a great proportion of strong CB+ cells located in outer layers with ectopic localization (inner molecular layers) (pattern 1) in TLE+D (Figures 2(f)–2(i)).

**Figure 3:**
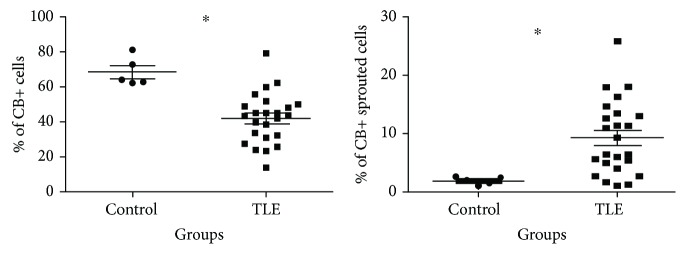
Semiquantitative analysis of dentate gyrus CB immunoreactivity observed in TLE patients and postmortem controls. Postmortem controls showed a significant higher number of CB+ cells, with a significant reduction of CB+ sprouted cells comparing with TLE patients (^∗^Student's *t*-test, *p* < 0.05).

The CB semiquantitative measurements (% of CB+ cell counting and % of CB+-sprouted cells) were compared between the TLE samples and postmortem controls using Student's *t*-test (normal distribution, Shapiro-Wilk test > 0.05) and/or Mann-Whitney test (Shapiro-Wilk test < 0.05). TLE samples showed a reduction in the percentage of CB+ cells by field (20x) (*x* = 41.96, SD = 14.47) compared with controls (*x* = 68.52, SD = 8.25) (*t* = −3.93, *p* = 0.001) (Student's *t*-test). TLE samples showed a higher percentage of cells with soma and dendritic CB+ expression (sprouted cells), TLE (mean rank = 17.38, *x* = 9.08, SD = 6.48) compared with controls (mean rank = 3.6, *x* = 0.78, SD = 0.33) (*U* = 3, *p* = 0.001) (Mann-Whitney), and longer CB+ dendritic processes (*μ*m), TLE (mean rank = 16.92, *x* = 10.91, SD = 7.50) versus controls (mean rank = 5.8, *x* = 3.96, SD = 1.50) (*U* = 14, *p* = 0.005) (Mann-Whitney) (Figure 3).

**Figure 4:**
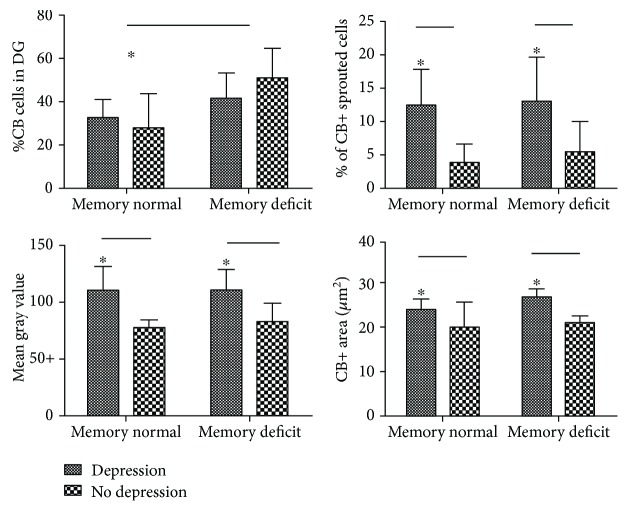
Patients with normal memory showed a lower counting of CB+ cells comparing with TLE patients with memory deficits (^∗^*p* < 0.05). Depression factor was significantly associated with higher % of CB+ cells with positive dendritic processes (sprouted cells), a higher CB+ optical density (MGV), and a higher CB+ area (^∗^*p* < 0.05) (two-way ANOVA). No interactions were observed between factors.

In the second exploration, we studied the association of depression and memory deficits in TLE (*n* = 14) and in TLE+D (*n* = 12) cases, in relation to the CB semiquantitative findings (% of CB+ counting, % of CB-sprouted cells, MGV, CB+ area, and length of CB+-sprouted processes) using the two-way ANOVA (Shapiro-Wilk test *p* > 0.05). Depression factor was associated with a higher % of CB+ cells with CB dendritic expression (CB+-sprouted cells) (*F*(1, 20) = 11.81, *p* = 0.003, hp^2^ = 0.37), a higher CB+ area (*μ*m^2^) (*F*(1, 20) = 5.33, *p* = 0.032, hp^2^ = 0, 21), and a higher optical density (MGV) (*F*(1, 20) = 15.09, *p* = 0.001, hp^2^ = 0.43). Memory deficits were associated with a lower reduction of the % of CB+ cells comparing patients with normal memory (*F*(1, 20) = 7.68, *p* = 0.012, hp^2^ = 0.28). No significant differences were observed in the % of CB+ cells regarding depression (*F*(1, 20) = 0.145, *p* = 0.70, hp^2^ = 007) (Figure 4). No significant differences were observed comparing memory deficit with CB+ MGV (*F*(1, 20) = 0.12, *p* = 0.72, hp^2^ = 0.006), with % of cells with CB+ dendritic expression (*F*(1, 20) = 0.29, *p* = 0.59, hp^2^ = 0.014), and neither with CB+ area (*μ*m^2^) (*F*(1, 20) = 0.92, *p* = 0.35, hp^2^ = 0.04). No significant interactions were observed between the factors. Additionally, no parametric comparison between TLE+D and TLE was determined for the length of CB+ processes. Longer CB+ processes (*μ*m) were also more frequently observed in TLE+D patients (mean rank = 16.42, *x* = 14.68, SD = 8.55) as compared with TLE without depression (mean rank = 8.58, *x* = 7.1, SD = 3.6) (*U* = 25, *p* = 0.007) (Mann-Whitney).

Pearson correlations were determined between clinical variables of epilepsy including psychiatric and cognitive variables, with CB expression. Age of epilepsy onset inversely correlated with the length of CB sprouting (*r* = −0.48, *p* = 0.17) and the same tendency but no significant was observed between the age of epilepsy onset and the % of CB+-sprouted cells (*r* = −0.32, *p* = 0.13). Age of epilepsy onset and % of CB+ cells were correlated (*r* = 0.33, *p* = 1.11). The % of sprouted cells showed a high correlation with the length of CB+ sprouting (*r* = 0.76, *p* = 0.0.001), the reactive area (*r* = 0.70, *p* = 0001), and the MGV (*r* = 0.68, *p* = 0.0001).

**Figure 5:**
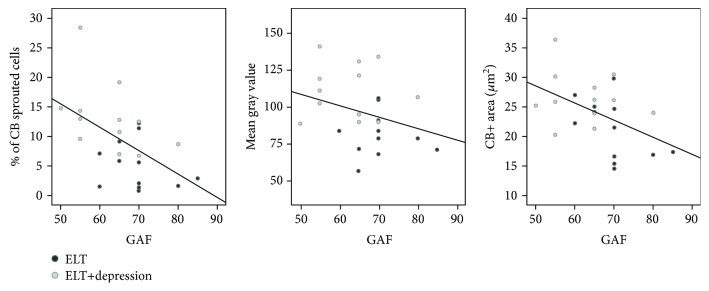
The GAF (global assessment of functioning, DSM-IV) inversely correlated with the % of CB sprouted cells (*r* = −0.52, *p* = 0.008), with the CB+ area (*r* = −0.46, *p* = 0.022), and with the optical density (mean gray value) (*r* = −0.33, *p* = 0.14).

In relation to psychiatric variables, the GAF scale (a quantitative measure of global functioning according to psychiatric morbidity) inversely correlated with the % of CB-sprouted cells (*r* = −0.52, *p* = 0.008) and with the CB+ surface area (*r* = −0.46, *p* = 0.022). No significant correlations were found with MGV (*r* = −0.33, *p* = 0.14) (Figure 5). The Beck inventory, which determines the current status of depression severity, showed no significant correlations between the CB+ surface area (*r* = 0.28, *p* = 0.27), the % of CB+ cells (*r* = 0.38, *p* = 0.13), the MGV (*r* = 0.395, *p* = 0.130), and the % of CB+-sprouted cells (*r* = 0.28, *p* = 0.29) (only 16 patients completed this scale).

Regarding cognitive variables, the *z*-scores of verbal and visual memory tests were analyzed ipsilateral for the epileptic focus. In the cases with a right focus, visual memory was considered, while in the cases with a left focus, verbal memory was considered. No significant correlations were found between the *z*-scores and the CB+ area (*r* = −0.01, *p* = 0.93), the % of CB+ cells (*r* = 0.20, *p* = 0.34), the MGV (*r* = −0.27, *p* = 0.212), and the % of CB+-sprouted cells (*r* = −0.04, *p* = 0.84). A tendency but no significant inverse correlation was found between the Beck inventory scores and the *z*-scores of verbal and visual memory tests (*r* = −0.39, *p* = 0.13).

## 4. Discussion

In this preliminary study, we found an important reduction of CB+ cell counting in dentate gyrus from TLE patients comparing controls similarly to other authors [27, 28, 32–34]. Additionally, we observed a differential pattern of CB immunoreactivity with a higher proportion of larger and stronger CB+ cells with sprouting processes projecting into the molecular layer in TLE patients with comorbid depression, especially in the most severe depressive patients with lower GAF scores. Comparable to these features, Ábrahám et al. described strong CB+ cells located at the outer layers of dentate gyrus in patients with TLE [34]; then, Martinian et al. described the pattern 1 of CB immunoreactivity characterized by a reduction of CB+ granular cells predominantly among basal layers with a great proportion of strong CB+ cells located in outer layers [33]. Furthermore, Thom described the CB immunoreactive pattern with the sprouting of CB+ granular cells, as a common occurrence in HS, and highlighted the importance of granular cell pathology to understand epilepsy comorbidities [17]. This pattern 1 was associated with a major extent of granular cell dispersion and with a higher mossy fiber sprouting which are signs that may indicate local higher hyperexcitability [17, 33, 48]. Hyperexcitability has been considered a factor that might enhance depression behavior in animal models of epilepsy and depression [3, 9], and Kandratavicius et al. found an enhanced mossy fiber sprouting using Neo-Timm staining in patients with epilepsy and comorbid depression [49].

It has been proposed that disrupted granule cell neuroplasticity in epilepsy may contribute to both hyperexcitability and neuropsychiatric illness favoring to behavioral and cognitive alterations [3, 9, 10, 11, 49]. The acute epileptogenic insult increases the production of new formed cells with aberrant sprouting, abnormal maturation, and ectopic integration into dentate gyrus layers contributing to hyperexcitability [9, 50]. However, this initial event followed by chronic epilepsy instauration (recurrent seizures) may affect cell survival resulting in a reduced number of mature adult neurons at the end of the process [12, 18, 19, 23, 25, 26], which may also contribute to enhance dentate gyrus excitability [51]. It has been recently reported that mature cells activate local GABAergic circuits favoring cellular inhibition; indeed, the low grade of maturity of granular cells may contribute to reduce hippocampal excitability [51, 52].

CB expression in dentate gyrus granular cells is considered a marker of granular cell maturity; thus, alterations in CB expression patterns may indicate a dysmaturation process [17, 29, 33]; however, the specific role of CB in granular cells is still controversial. CB is a calcium-binding protein that is usually located in neocortex in GABAergic interneurons, but in dentate gyrus, CB predominates in glutamatergic granular cells and is expressed in the later stages of hippocampal neurogenesis denoting the initiation point of synaptogenesis (first step of the trisynaptic circuit receiving the perforant path from the entorhinal cortex) [29, 50]. CB content in granular cells may influence the cell survival capacity and was proposed as a neuromodulator marker essential to hippocampal well functioning [33]; nevertheless, the physiological role of CB in granular cells has not been clearly determined. In addition, several studies reported a neuroprotective activity through buffering intracellular calcium; these findings were mostly determined in the cerebral cortex, and on the contrary, the decreasing CB might protect these cells from excitotoxicity in granular cells of dentate gyrus contributing to the resistance of survival [53]. In this line, it has been observed that an overexpression of CB in DG neurons may disrupts LTP (long-term potentiation) affecting the CB homeostatic role in synaptic plasticity [54]. Similarly, an overexpression of CB in dentate gyrus layers increased mossy fiber excitatory postsynaptic potentials and impaired spatial navigation in experimental models [36].

Overall, these findings have supported the theory that bidirectional facilitation mechanisms are involved between hyperexcitabilities due to seizures and depression [3, 55, 56]. In chronic epilepsy, recurrent neuronal hyperexcitability may reduce neuroplasticity and neurogenesis contributing to depressive behavior probably enhancing stress vulnerability mechanisms [9, 10, 19, 57, 58]. Additionally, chronic stress and glucocorticoids affect dentate gyrus layer functioning, enhancing neuronal excitability and reducing hippocampal neuroplasticity and neurogenesis favoring both depression and hyperexcitability [59–63]. Similar to epilepsy, depression, and chronic stress, cause plastic remodeling in the hippocampal networks inducing a loss of dendritic spines at CA3 layers and reducing NG in dentate gyrus [64]. However, this process is reversible and is principally mediated by glucocorticoids acting throughout mineralocorticoid/glucocorticoid receptors and glutamate [61, 65]. These mechanisms have been involved in the hippocampal volume loss associated to major depression [62]. Similar to epilepsy, in chronic stress and depression models, cortisol and excitatory amino acids play a principal role in exerting a potential deleterious effect in the brain inducing granular cell hyperexcitability, lower neuroplasticity, and higher neuroinflammation response [66–70]. According to these findings, stress response system activation during patient's life may potentiate epileptic hippocampal pathogenic mechanisms and dentate gyrus hyperexcitability facilitating and enhancing stress vulnerability leading to depression.

Regarding memory, a CB overexpression has been associated with lower hippocampal-dependent memory [36] and, on the other side, CB loss has been also observed after chronic stress and was associated with memory deficits in experimental models [54]. Furthermore, the reduction of mature granular cells in dentate gyrus has been related to a poorest cognitive performance in epileptic patients [71]. Additionally, studies using human cells isolated from surgical material of patients with resistant epilepsy found a low proliferative capacity in subjects with more severe learning and memory deficits [12]. A few studies determined CB dentate gyrus expression and cognitive performance in patients with epilepsy [32, 33] showing controversial results. Martinian et al. qualitatively described the three different patterns of CB immunoreactivity mentioned before, but they did not found any differences comparing the patterns of CB expression in relation to memory deficits [33]; however, Karádi et al. found a correlation between CB granular cell loss and verbal scores but not with visual memory scores [32]. The controversial results may be due to the different methods used for clustering data. In this study, the CB overexpression pattern with a higher CB sprouting was associated with depression while the reduction of CB counting was higher in patients with normal episodic memory (both hippocampal dependent memories verbal and visual were normal). Further analysis should be made to confirm these controversial findings regarding memory.

Limitations of this study must be mentioned. First, this is a small and exploratory study; further and larger investigations using other biomarkers and double immune stains must be performed to confirm the mechanisms involved in these preliminary findings. Furthermore, depression and memory deficits were only analyzed in patients with TLE under chronic treatment with different kinds of drugs including antidepressants and antiepileptic treatments; indeed, the conclusions of this study must be referred to this special population. In this study, we did not analyze the postsurgical outcome, but this will be an interesting approach for future studies.

## 5. Conclusions

In this exploratory study, comorbid depression was associated with a different pattern of CB cell loss in dentate gyrus with CB overexpression among outer granular cell layers. These changes may indicate granular cell dysmaturation associated to the epileptic hyperexcitability phenomena, supporting the bidirectional relationship theory described between depression and epilepsy. Nevertheless, we cannot know if these are adaptive changes and the functional meaning and/or the functional consequences of these findings. Granular cell pathology constitutes a possible common basis to understand the neurobiological mechanisms involved in the psychiatric comorbidities of epilepsy. Further and larger investigations should be made to confirm these preliminary findings.

## Figures and Tables

**Table 1:** Clinical data of patients with TLE with and without comorbid depression (*n* = 24).

Case number	Sex	Age of seizure onset	Age of surgery	Time of epilepsy duration	RMN	Type of depression DSM-IV	Suicide attempts	Psychopharms received	Antiepileptic treatment	GAF	Beck inventory	Memory deficits	Hippocampal sclerosis type	Dentate gyrus pathology	% of CB-IR-sprouted cells
1	M	9	42	33	RHS	No	No	No	DFH, TPM	65	n/a	GMD	I	Yes	∗∗
2	M	18	24	6	RHS	No	No	No	CZP, CL	70	n/a	ViMD	I	Yes	∗∗
3	F	2	28	26	RHS	RMD+P	No	AD, RP	CZP, VPA, CL	55	n/a	ViMD	I	Yes	∗∗∗
4	F	8	24	16	LHS	MD+P	Yes	FLX, RP	CBZ, LMT	70	n/a	GMD	I	Yes	∗∗∗∗
5	F	23	41	18	LHS	No	No	No	TPM, CBZ, CL	60	n/a	VMD	I	No	∗
6	F	17	46	29	RHS	No	No	No	CBZ, TPM	70	n/a	ViMD	I	No	∗
7	F	32	47	15	RHS	No	No	No	CBZ, CL	70	n/a	ViMD	I	Yes	∗
8	F	3	22	19	RHS	RMD	Yes	CT	LMT, CBZ	65	n/a	Normal	I	No	∗∗∗
9	F	13	36	23	RHS	RMD+D	No	AD	CZP, CL	55	23	Normal	I	Yes	∗∗∗∗
10	F	7	22	15	RHS	No	No	No	CBZ, TPM	70	8	ViMD	I	Yes	∗
11	F	15	18	3	RHS	No	No	No	CBZ, LTG	80	13	Normal	I	Yes	∗∗
12	F	1	41	40	LHS	No	No	No	DFH, CBZ, LVT	70	10	Normal	I	Yes	∗
13	M	12	22	10	RHS	No	No	No	DFH, LMT, CL	60	6	ViMD	I	Yes	∗∗∗∗
14	M	1	40	39	LHS	No	No	No	VPA, CL	70	4	Normal	I	Yes	∗∗∗
15	F	11	29	18	LHS	No	No	No	CBZ, CL	65	6	ViMD	I	Yes	∗
16	M	25	37	12	LHS	No	No	No	CBZ, TPM, CL	85	8	ViMD	II	Yes	∗∗
17	F	10	24	14	LHS	RMD	No	ST	LMT, CBZ	65	21	GMD	I	Yes	∗∗∗∗
18	M	6	17	11	RHS	MD	No	No	OXC, LVT, CL	70	29	VMD	I	Yes	∗∗
19	F	18	24	6	RHS	MD	No	No	CBZ, CL	80	9	VMD	I	Yes	∗
20	F	1	39	38	LHS	MD+D	No	No	LMT, LCS	65	5	Normal	I	Yes	∗∗∗∗
21	F	17	52	35	LHS	RMD+D	No	AD	LMT, LVT, CL	55	24	Normal	I	Yes	∗∗∗
22	F	5	18	13	LHS	D	No	No	OXC, CL	65	1	VMD	II	No	∗∗
23	M	8	25	17	LHS	RMD+D	No	No	TPM, CBZ, FT	55	20	GMD	I	Yes	∗∗∗∗
24	F	2	34	34	LHS	RMD+P	Yes	ST, OLN, RP, LSD	VPA, FB, CBZ	50	22	GMD	I	Yes	∗∗∗

GAF: Global Assessment of Functioning (DSM-IV); RMD: recurrent major depression; MD: major depression; D: dysthymia; n/a: not available data; P: psychotic symptoms associated with depression; PPT: psychopharmacological treatment received during life; AD: antidepressants (not determined which one); FLX: fluoxetine; CT: citalopram; ST: sertraline; RP: risperidone; LSD: lurasidone; OLZ: olanzapine; AED: antiepileptic drug treatment; DPH: diphenylhydantoin; TPM: topiramate; CBP: carbamazepine; CL: clonazepam; VPA: divalproate; LMT: lamotrigine; LVT: levetiracetam; FB: phenobarbital; LCZ: lacosamide; HS: hippocampal sclerosis—type I (CA1+CA4 predominantly affected) and type II (CA1 predominantly affected); VMD: verbal memory deficit, ViMD: visuospatial memory deficit; GMD: global memory deficit. Dentate gyrus pathology according to Blümcke et al. [13]. The % CB+ sprouted cells: ∗ is <10, ∗∗ is ≤15 but >10% ∗∗∗ is ≤20 but >15%, and ∗∗∗∗ is >20% of CB IR.

## Data Availability

The data used to support the findings of this study are available from the corresponding author upon request.
